# Knockdown of interleukin-10 induces the redistribution of sigma1-receptor and increases the glutamate-dependent NADPH-oxidase activity in mouse brain neurons

**DOI:** 10.1186/s40659-015-0048-1

**Published:** 2015-10-09

**Authors:** S. Koriauli, N. Natsvlishvili, T. Barbakadze, D. Mikeladze

**Affiliations:** Ilia State University, 3/5 K. Cholokashvili Ave., 0162 Tbilisi, Georgia; I. Beritashvili Center of Experimental Biomedicine, 14 Gotua Str., Tbilisi, Georgia

**Keywords:** Interleukin-10, NMDA-receptor, NR2B subunit, NADPH-oxidase, Rac1, BiP, SigR1, ER stress, Neurodegeneration, Neuroinflammation

## Abstract

**Background:**

In the central nervous system, interleukin-10 (IL-10) provides trophic and survival effects directly on neurons, modulates neurite plasticity, and has a pivotal importance in the neuronal regeneration in neurodegenerative and neuroinflammatory conditions. This cytokine is primarily produced by glial cells and has beneficial effects on the neuronal viability. However, the mechanisms of IL-10-elicited neuroprotection are not clear.

**Results:**

Membrane preparations, isolated from wild-type (Wt) and IL-10 knockout (KO) mice brain were used in this study. It has been shown that compared to wild-type mice, in IL-10 KO mice brain, the amount of immunoglobulin binding protein (BiP) is greatly increased, whereas the content of sigma receptor-1 (SigR1) is not changed significantly. Co-immunoprecipitation experiments have shown that the association of SigR1 with small GTPase Rac1 (Ras-related C3 botulinum toxin substrate 1), NR2B subunit of NMDA-receptor (NMDAR) and inositol-3-phosphate receptor (IP3R) is higher in the IL-10 KO mice brain than in the Wt mice brain. Besides, we have found that either glutamate or sigma ligands, separately or together, do not change glutamate-induced NADPH-oxidase (NOX) activity in Wt-type mice brain membrane preparations, whereas in IL-10 KO mice high concentration of glutamate markedly increases the NOX-dependent production of reactive oxygen species (ROS). Glutamate-dependent ROS production was decreased to the normal levels by the action of sigma-agonists.

**Conclusions:**

It has been concluded that IL-10 deprivation, at least in part, can lead to the induction of ER-stress, which causes BiP expression and SigR1 redistribution between components of endoplasmic reticulum (ER) and plasma membrane. Moreover, IL-10 deficiency can change the specific organization of NMDAR, increasing the surface expression of SigR1-sensitive NR2B-containing NMDAR. In these conditions, glutamate-dependent ROS production is greatly increased leading to the initiation of apoptosis. In this circumstances, sigma-ligands could play a preventive role against NMDA receptor-mediated excitotoxicity.

## Background

Interleukin-10 (IL-10), an anti-inflammatory cytokine, modulates neurite plasticity, and has a pivotal importance in neuroregeneration. [1–4]. IL-10 acts as a growth factor [5], regulates neurogenesis in the normal adult brain, promotes axonal outgrowth [4], and provides trophic and survival effects against glutamate-dependent excitotoxicity [6]. This cytokine induces an increase in neuronal survival that correlates with changes in the microglial phenotype, density, and microglial clustering [7]. IL-10 produced by microglia reduces neuroinflammation and increases neuronal survival that are mediated by inhibition of NADPH oxidase activity [8]. Most neural effects of IL-10 are mediated by direct interaction with the neuronal IL-10 receptor [6].

IL-10 can change long-term potentiation, suggesting that a target of the cytokine is the system participating in glutamatergic neurotransmission [9]. An IL-10 deficiency reduces the expression of mGlu-receptor 1a/b, decreases the sensitivity of NMDA-receptor to the polyamines and changes the glutamate-dependent production of nitric oxide [10]. In addition to an activation of the canonical signaling pathways, this cytokine is capable of exerting the rapid neuroprotective effects through inhibition of inositol-3-phosphate receptor (IP3R)-sensitive Ca^2+^ release from internal stores after repeated NMDA receptor stimulation [11]. Thus, deprivation of IL-10, through modification of IP3R may initiate ER-stress and uncontrolled release of Ca^2+^, which is characterized by a redistribution of ER-responses chaperones and increased production of ROS [12]. In these perturbations, NADPH oxidase may play the significant role, as a primary source of ROS produced in the result of NMDA-receptor overactivation [13]. According to this hypothesis, here we have found that in mice IL-10 deficiency induces the ER stress in the neurons and causes a redistribution of the ER stress response proteins—BiP and sigma receptor-1 (SigR1). In IL-10 KO mice brain the association of SigR1 with IP3R and Rac1 (Ras-related C3 botulinum toxin substrate 1), as well as with NR2B subunit of NMDAR is increased that leads to the glutamate-dependent and SigR1-sensitive activation of NADPH-oxidase (NOX). These data suggest that IL-10 can decrease the NMDAR-dependent production of ROS during the ER-stress through modulation of SigR1 that may be important for understanding the mechanisms of neuroprotective action of IL-10.

## Results and discussion

**Fig. 1:**
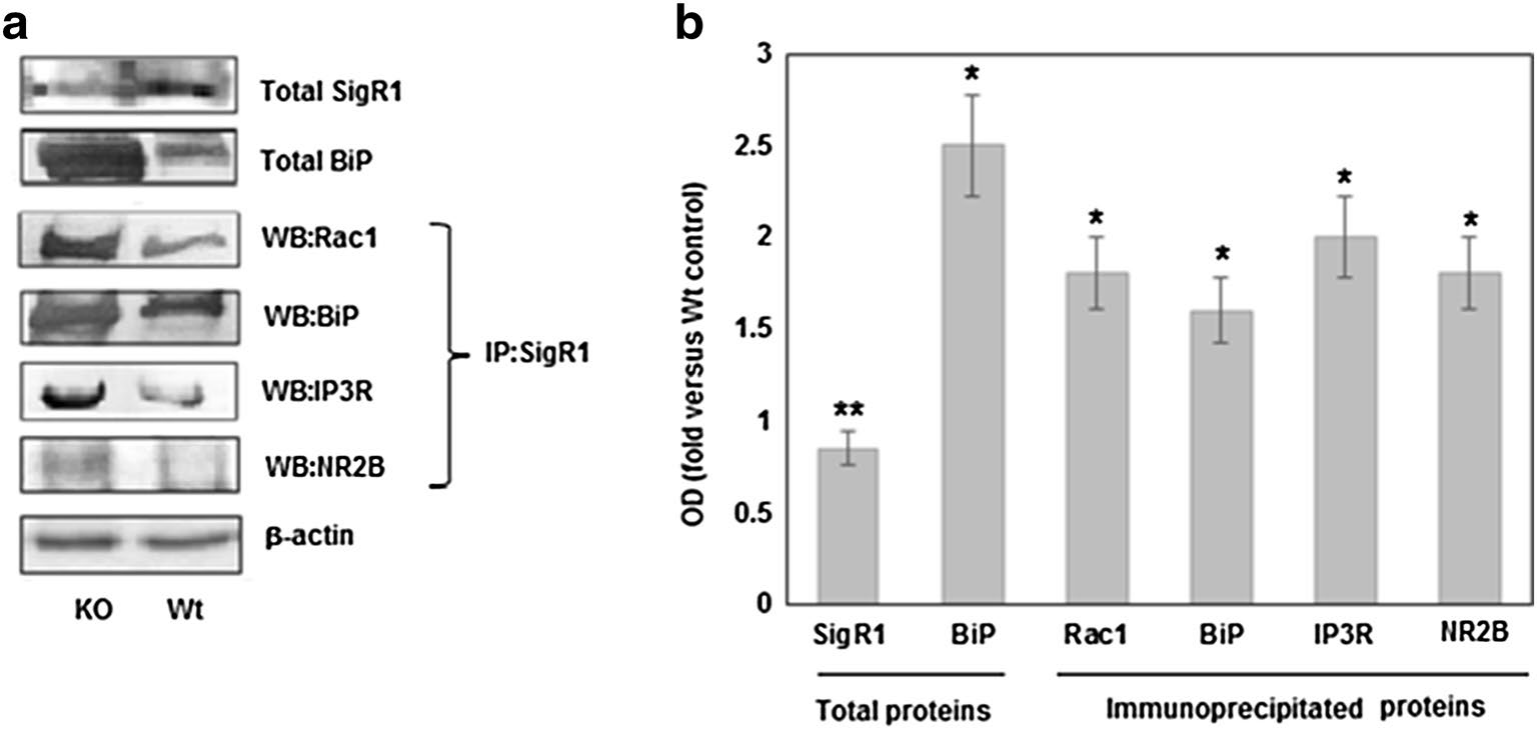
Western blot analysis of SigR1, BiP, Rac1, IP3R and NR2B in membrane preparations of KO and Wt mice brains. Total SigR1 and BiP were analyzed directly in membrane preparations. For the co-immunoprecipitation experiments solubilized membrane preparation were immunoprecipitated with anti-Sig1R, the immunoprecipitate was subjected to SDS-PAGE and transferred. **a** The *blots* were probed to Western analysis with antibodies against Rac1 (Wb: Rac1), BiP (Wb: BiP), IP3R (Wb: IP3R) and NR2B (Wb: NR2B). *β*-Actin was also visualized by Western blotting to confirm equal loading of the fractions. The blot is representative of four similar experiments. **b** Quantification of blots shown in B; n = 4. *P < 0.05, compared with control (Wt) levels. **P > 0.05, compared with control (Wt) levels

The endoplasmic reticulum ER stress is crucial in the pathogenesis of various neurodegenerative diseases [14]. It has been shown that IL-10, in epithelial cells, together with NADPH oxidase (NOX) alleviates the ER stress and can reduce a sensitivity to inflammation [15]. To study the possible involvement of the IL-10 in the ER stress in the neurons we tested the content of two chaperone proteins—BiP and SigR1 in the IL-10 KO mice brain. We have found that the amount of BiP is greatly increased in the KO mice brain, whereas the content of SigR1 is not changed significantly (Fig. 1a, b).

It is well established that in resting conditions, the SigR1 forms a complex with the BiP at the MAM [16]. During ER stress or via ligand stimulation, sigma-1 receptors dissociate from BiP and modulate the activity of IP3R. The stabilization of IP3R3 by Sig-1Rs ensures the proper Ca2+ influx into mitochondria, leading to the enhancement of ATP production [17]. To evaluate the association of SigR1 with BiP and IP3R, the coimmunoprecipitation experiments were performed. Extracts from membrane preparations were immunoprecipitated with agarose-conjugated anti-Sig1R antibodies and eluted proteins were subjected to Western blot analysis (Fig. 1a). We have found that the association of SigR1 with BiP and IP3R is higher in the IL-10 KO mice brain than in the Wt mice brain.

Rac1 pathway is involved in stress signaling through the formation of ROS [18]. Our recent investigation has shown that SigR1 could directly interact with Rac1 through multiprotein complex IP3R/Sig1R/Bcl-2/Rac1 [19]. Thus, in the next, we examined the association of Rac1 with SigR1 in the IL-10 KO mice brain. Our results have shown that binding of Rac1 to SigR1 increased in IL-10-deficient mice brain. (Fig. 1a, b). These findings suggest that in the IL-10 KO mice brain the complexation of SigR1 with IP3R and Rac1 is risen that can be involved in protective or adaptive responses to IL-10 deficiency.

Glutamate exposure may increase Ca2+ influx into neurons and may stimulate the generation of oxidative/nitrosative species that damages the cell [20]. Besides mitochondria, important ROS producers in the cells are NADPH-oxidases [21] that could be regulated by Rac1 [22]. Plasma membrane-bound Rac1-NADPH oxidase complex may play an essential role in the glutamate-induced apoptotic cell death through increased production of ROS [23]. Rac activity and elevation of ROS has been linked to ER stress, which could, in turn, induces the perturbations in chaperon’s machinery [24, 25]. Thus, in the next, we determined the NADPH-oxidase activity in the presence of glutamate and sigma ligands.

**Fig. 2:**
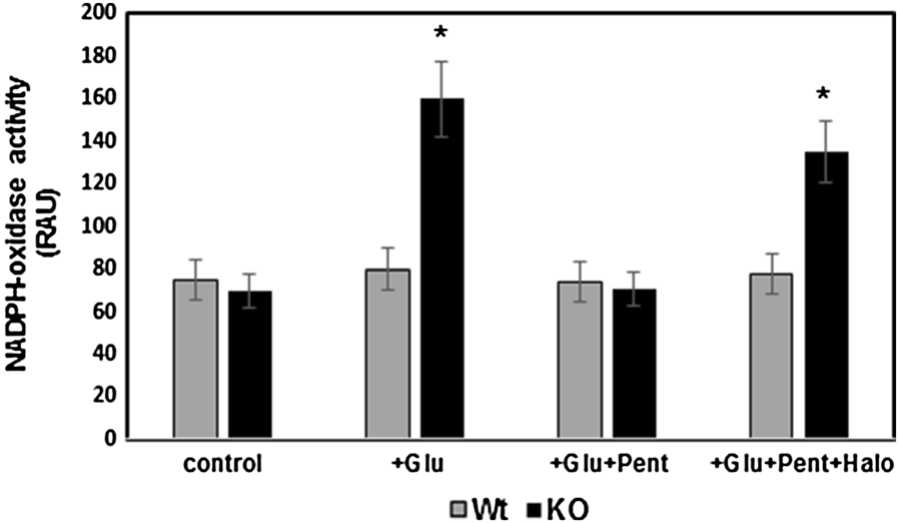
Effects of glutamate, (+)-pentazocine, and haloperidol on the NADPH oxidase activity in membrane preparations of KO and Wt mice brains. Membrane preparations were incubated with glutamate (Glu), haloperidol (Halo), with (+)-pentazocine (Pent) or with glutamate and both sigma-ligands (Glu + Halo + Pent) and NADPH oxidase activity determined as described in “Methods”. Data represented are mean ± SEM of results from four separate experiments performed in duplicate. *P < 0.05, *t* test compared with corresponding control (c)

We have found that either glutamate or sigma ligands, separately or together, do not change NADPH-oxidase activity in Wt-type mice membrane preparations. However, in IL-10 KO mice glutamate markedly increases the NADPH-dependent production of ROS (Fig. 2). Glutamate-induced NADPH-oxidase activity was decreased in the presence of sigma-agonist—(+)-pentazocine, whereas haloperidol (sigma-antagonist) eliminates the action of (+)-pentazocine. The same results were obtained after addition of 4-PPBP (sigma 1 agonist) and 1 μM BD1063 (sigma1 antagonist) (data not shown). These data suggest that in IL-10 KO mice glutamate could induce ROS generation through the acceleration of NADPH-oxidase in the brain, and sigma agonists alleviate this effect. It is interesting to note, that in the bovine brain mitochondria sigma-agonists increased NOX activity [19], which is apparently due to the different intracellular organization of the macromolecular complex of SigR1.

Because activation of SigR1 in the ER leads to the translocation of SigR1 from the mitochondria-associated membrane (MAM) to the plasma membrane, we propose that NMDAR-associated SigR1 may mediate this anti-oxidative effect of sigma-agonists. Translocation of SigR1 and enhanced interaction between Sig1Rs and NR2B subunits of NMDARs was observed after SigR1 activation [26]. These relocations could disrupt protein–protein interactions between NMDARs and neuronal nitric oxide synthase (nNOS), decreasing the production of nitric oxide (NO) [27]. nNOS-derived NO is the signaling molecule linking NMDA receptors to the activity of NOX and the association between NMDA and nNOS via PSD95 is required for the ROS production [28]. Thus, SigR1 through of NMDAR/PSD95/nNOS complex could down-regulated the glutamate-dependent production of ROS. To test this hypothesis, we determined the content of NMDAR in immunoprecipitated SigR1 preparation and found that a greater amount of NR2B subunit is associated with the SigR1 in KO mice brain (Fig. 1a, b). These data suggest that deficiency of IL-10 could change SigR1-dependent redistribution of NMDAR between ER/MAM and plasma membrane increasing SigR1-sensitive forms of NMDAR. IL-10 significantly increased neuronal synapse formation in the early development stage [29] and thus, deficiency of this cytokine could induce the abnormal organization of synaptic and extrasynaptic machinery. Our previous investigation has shown that the total level of the subunits of NMDAR (NR2A and NR2B), as well as the level of NMDAR-bound nNOS, are not changed in KO mice. Despite this, NMDAR from KO mice has different ligand-binding properties and exhibits significantly lower sensitivity to polyamines [10]. These data suggests that IL-10 deficient mice are comprising splicing isoforms of NMDAR, which may have higher sensitivity to the protective actions of SigR1.

In summary, our results showed that IL-10 deprivation, at least in part, can lead to the induction of ER stress, which causes expression of BiP and redistribution of SigR1 between components of ER and plasma membrane. Moreover, IL-10 deficiency can change the specific organization of NMDAR, increasing the surface expression of SigR1-sensitive NR2B-containing-NMDAR. In these conditions, glutamate-dependent ROS production is greatly increased leading to the initiation of apoptosis. In this circumstances, sigma-ligands could play a preventive role against NMDA receptor-mediated excitotoxicity.

## Methods

### Animals and social conditions

All procedures were conducted by the guidelines of Ilia State University Research Project Ethics Commission. Male C57BL/6 J wild-type and IL-10-deficient C57BL/6 J mice (mice homozygous for the IL10tm1Cgn targeted mutation, 8–12 weeks of age) were purchased from Jackson Laboratories (Bar Harbor, ME, USA) and provided by the Institute of Medical Biotechnology of Tbilisi State Medical University (Tbilisi, Georgia). These IL-10 mutant mice are viable and fertile under specific pathogen-free conditions. IL-10-deficient phenotype is associated with altered lymphocyte and myeloid profiles, elevated serum amyloid. A levels, altered responses to inflammatory or autoimmune stimuli, increased depressive-like behavior [30] and decreased neuroprotection [31].

### Preparation of membrane fractions

The brain cortex was removed and gently homogenized in isolation buffer (50 mM HEPES, pH 7.5, 125 mM NaCl, 2 mM KCl, 100 mM sucrose, 1,8 mM CaCl_2_, 1 mM MgCl_2_, 5 U/mL aprotinin). Homogenate was centrifuged at 1250×*g* for 5 min. Obtained supernatant was immediately centrifuged at 21,000×*g* for 20 min and membrane fractions were used for further experiments.

### Coimmunoprecipitation and immunobloting

Membrane fractions were resuspended in ice-cold solubilization buffer (20 mM Tris–HCl pH 8.0, 137 mM NaCl, 10 % glycerol, 1 % Triton X-100, 2 mM EDTA), incubated for 30 min at 4 °C. The unsolubilized material was removed by centrifugation (60 min at 20,000×*g*). Anti-SigR1 antibody (rabbit polyclonal OPRS1, Abcam 53852, UK) and prewashed Protein A/G-agarose resin (Pierce) were added to the supernatant at 5 μg of antibody per 400 μg of protein and incubated overnight at 4 °C. After washing (12,000×*g*, 20 min), the protein A/G-Agarose pellets were resuspended in 100 mM glycine, pH 3.0, for 10 s, and then a pretitrated volume of 1.0 M Tris, pH 9.5, was added to adjust the pH to 7.4. Protein complexes in the supernatants (1000×*g*, 12 min) were then analyzed by Western blotting.

For immunoblotting experiments, 50 μg of protein was separated by SDS–polyacrylamide gel electrophoresis and transferred to nitrocellulose sheets. After blocking with blocking buffer (5 % bovine serum albumin, 0.05 % Tween 20 in Tris–HCl-buffered saline), the sheets were incubated either with BiP rabbit polyclonal BiP antibody (Abcam, UK), with Rac1 rabbit polyclonal antibody (Santa Cruz, USA), with rabbit monoclonal inositol 1,4,5-trisphosphate receptor (IP3R) antibody, (Abcam, UK), or with goat polyclonal NR2B glutamate receptor (ε2) antibody (Santa Cruz, USA). As a loading control, β-actin expression levels were measured by goat polyclonal anti-actin antibody (Santa Cruz, USA). The nitrocellulose membranes were washed three times with wash buffer (150 mM NaCl, 10 mM Tris–HCl, pH 7.0, and 0.05 % Tween 20) containing 5 % skim milk. And then incubated with horseradish peroxidase-conjugated goat-anti-rabbit or rabbit-anti mouse-IgG antibody. Labeled bands were visualized using enhanced chemiluminescence (Amersham, California, USA) and analyzed by densitometric scanning. The content of proteins was quantified by the intensity of the bands, which is linear to the quantity of samples applied to the gel. The protein levels were normalized against β-actin intensity.

Protein concentration was determined using a dye-binding method (Bio-Rad).

### NADPH oxidase activity

NADPH oxidase activity was measured in mice brain according to O’Brien et al. 2009 [32]. The membrane fractions were resuspended in 50 mM phosphate buffer at pH 7.4. Paired assays were conducted by incubation of 20–60 μg of membrane proteins with 10 mM phosphate buffer, 130 mM NaCl, 1 mM EGTA, 10 μM flavin adenine dinucleotide (FAD), 2 mM NaN3, 50 μM oxidized cytochrome *c*, and 10 μM GppNHp (Guanosine 5′-[β,γ-imido]triphosphate). In some experiments 100 μM glutamate, 1 μM (+)-pentazocine, 1 μM haloperidol, 1 μM 4-PPBP or 1 μM BD1063 were added. One reaction of each pair contained 200 units of superoxide dismutase (SOD; Sigma–Aldrich). Reactions were initiated by the addition of NADPH to a final concentration of 200 μM. After 1 h, activity was measured as the SOD-inhibitable increase in absorbance at 550 nm. Results of the NOX activity are expressed as the reduced cytochrome c per milligram protein per min as relative absorbance units at 550 nm (RAU at 550 nm).

### Statistical analysis

All experiments were performed in triplicates and repeated at least twice. Results are expressed as mean ± the standard error of the mean (SEM). For statistical analysis, a *t* test was used. A difference between experimental groups was considered to be significant when P < 0.05.

## Abbreviations

IL-10: Interleukin-10
Wt: Wild type mice
KO: Knockout mice
BiP: Immunoglobulin binding protein
SigR1: Sigma receptor-1
IP3R: Inositol-3-phosphate receptor
NOX: NADPH-oxidase
ROS: Reactive oxygen species
ER: Endoplasmic reticulum
Rac1: Ras-related C3 botulinum toxin substrate 1
MAM: Mitochondria-associated membrane
nNOS: Neuronal nitric oxide synthase
NO: Nitric oxide
PSD95: Postsynaptic density protein 95
